# New Insights on Steroid Biotechnology

**DOI:** 10.3389/fmicb.2018.00958

**Published:** 2018-05-15

**Authors:** Lorena Fernández-Cabezón, Beatriz Galán, José L. García

**Affiliations:** ^1^Department of Environmental Biology, Centro de Investigaciones Biológicas, Consejo Superior de Investigaciones Científicas, Madrid, Spain; ^2^Novo Nordisk Foundation Center for Biosustainability, Technical University of Denmark, Lyngby, Denmark

**Keywords:** steroid, pharmaceuticals, actinobacteria, microbial cell factory, metabolic engineering, systems biology

## Abstract

Nowadays steroid manufacturing occupies a prominent place in the pharmaceutical industry with an annual global market over $10 billion. The synthesis of steroidal active pharmaceutical ingredients (APIs) such as sex hormones (estrogens, androgens, and progestogens) and corticosteroids is currently performed by a combination of microbiological and chemical processes. Several mycobacterial strains capable of naturally metabolizing sterols (e.g., cholesterol, phytosterols) are used as biocatalysts to transform phytosterols into steroidal intermediates (synthons), which are subsequently used as key precursors to produce steroidal APIs in chemical processes. These synthons can also be modified by other microbial strains capable of introducing regio- and/or stereospecific modifications (functionalization) into steroidal molecules. Most of the industrial microbial strains currently available have been improved through traditional technologies based on physicochemical mutagenesis and selection processes. Surprisingly, Synthetic Biology and Systems Biology approaches have hardly been applied for this purpose. This review attempts to highlight the most relevant research on Steroid Biotechnology carried out in last decades, focusing specially on those works based on recombinant DNA technologies, as well as outlining trends and future perspectives. In addition, the need to construct new microbial cell factories (MCF) to design more robust and bio-sustainable bioprocesses with the ultimate aim of producing steroids à *la carte* is discussed.

## Introduction

**Figure 1:**
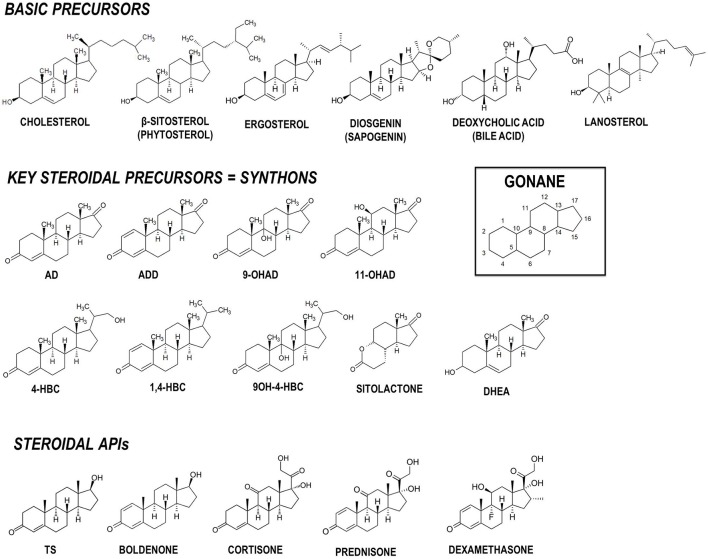
Chemical structures of relevant steroidal compounds containing the gonane tetracyclic skeleton. Structures of basic precursors, key intermediates (synthons) and active pharmaceutical ingredients (APIs) are shown. AD, 4-androstene-3,17-dione; ADD, 1,4-androstadiene-3,17-dione; 9OH-AD, 9α-hydroxy-4-androstene-3,17-dione; 4-HBC, 22-hydroxy-23,24-bisnorchol-4-ene-3-one; 1,4-HBC, 22-hydroxy-23,24-bisnorchol-1,4-diene-3-one; 9OH-4-HBC, 9,22-dihydroxy-23,24-bisnorchol-4-ene-3-one; 11OH-AD, 11α-hydroxy-4-androstene-3,17-dione; DHEA, 3β-hydroxy-5-androstene-7-one, dehydroepiandrosterone; TS, testosterone.

Steroids are a family of terpenoid lipids widely distributed in nature that present a relatively rigid common structure named gonane formed by four fused alicyclic rings (Figure 1). The oxidation state of the steroid nucleus rings and the presence of different attached functional groups determine the particular biological properties of each steroidic molecule, i.e., its biological function (Lednicer, 2011). Steroidal compounds play important biological roles in different organisms, including cell membrane stabilization and regulation of relevant cellular processes such as cell proliferation and tissue differentiation. For instance, sterols having a hydroxyl group at C-3 and a side-chain of eight or more carbon atoms at C-17 act as stabilizing agents in cell membranes in animals (cholesterol), plants (phytosterols), yeasts and fungi (ergosterol), and certain bacteria (e.g., lanosterol) (Figure 1; Pearson et al., 2003; Lamb et al., 2007; Nelson and Cox, 2012; Wei et al., 2016). Steroidal compounds are also relevant carbon and energy sources for different bacteria (Galán et al., 2017a) and appear participate in various cellular signaling mechanisms.

Steroid-based drugs present a broad range of therapeutic applications and represent the highest marketed category of pharmaceuticals after antibiotics with an annual production of more than one million tons. According to Tong and Dong (2009) the annual global market for steroid-containing products exceeds $10 billion with about 300 steroid drugs approved to date (Sedlaczek and Leland, 1988; Demain, 1992; Bureik and Bernhardt, 2007; Donova, 2017). Nevertheless the recent Global Steroids-Corticosteroid Market Research Report 2017 estimates a market size of 3,840 million $ in 2016 and it will reach 4,680 million $ by 2021 (Reports and Market, India). First studies on steroid synthesis took place in the early twentieth century and were notably increased in the 1950s due to the discovery of the pharmacological properties of progesterone and hydrocortisone. Steroidal active pharmaceutical ingredients (APIs) have been classically synthesized by chemical processes (Herráiz, 2017). However, the potential of microbial steroid biotransformation is known since several decades since its application offer a number of advantages over chemical synthesis: (i) regio- and/or stereospecific functionalization of molecules at positions not always available for chemical agents, (ii) multiple consecutive reactions carried out in a single operation step, (iii) more eco-friendly processes (i.e., mild reaction conditions, aqueous media). One of the first demonstrations of this potential was described with the production of cortisone. Traditionally, cortisone was synthesized from deoxycholic acid in a multi-step chemical process (31 steps) characterized by low mass yields (0.16%) and high economic costs (200 $/g, 1949). The incorporation of a biotransformation step with *Rhizopus arrhizus* ATCC 11145 and *Aspergillus niger* ATCC 9142 markedly reduced the number of required chemical steps (11 steps) and production costs (1$/g, 1979) of the industrial process (Carballeira et al., 2009). Similarly, other chemical steps have been replaced by microbial bioconversions in steroid synthesis processes in the last decades, leading to more competitive and robust industrial processes. For example, the steroid hormone testosterone (TS) is chemically synthesized from the steroidal intermediate 4-androstene-3,17-dione (AD), which is previously obtained from natural sterols by microbial biotransformation (Fernández-Cabezón et al., 2017a).

In this context, this review aims to compile the most relevant microbial bioprocesses of synthesis and/or functionalization of steroidal compounds described to date, focusing mainly on those designed in the light of the new metabolic engineering approaches. These bioprocesses, conventionally classified within the scope of White or Industrial Biotechnology, have been designed primarily by traditional approaches based on the isolation of microorganisms that produce a molecule of interest or that biocatalyze a particular process and their subsequent improvement through tedious processes of physical or chemical mutagenesis and selection. Nevertheless, in recent years, new bioprocesses have been also designed by recombinant DNA technology approaches, that open up new opportunities for the construction of more robust and versatile microbial cell factories (MCF) for the production of steroids à la carte.

**Figure 2:**
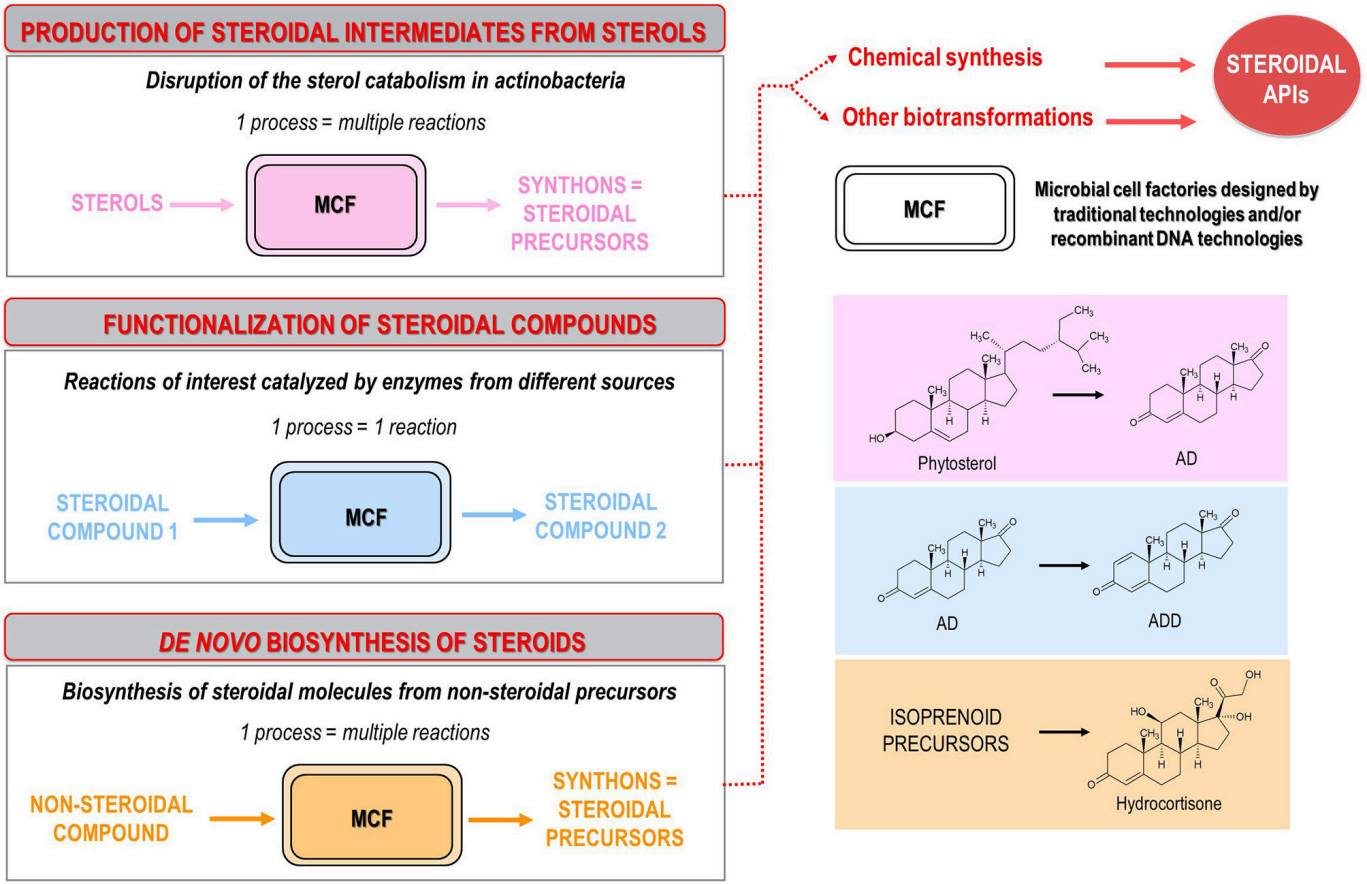
Classification of the microbial bioprocesses for steroid synthesis described to date: (i) production of steroidal intermediates from natural sterols (e.g., bioconversion of phytosterols into ADD); (ii) functionalization of steroidal molecules (e.g., bioconversion of AD into ADD); (iii) *de novo* biosynthesis of steroids (e.g., biosynthesis of hydrocortisone from non-steroidal substrates). Several microbial strains, isolated from natural sources and improved by conventional mutagenesis or designed by using recombinant DNA technologies, are used as microbial cell factories (MCF) to produce key steroidal intermediates (synthons). The resulting synthons are subsequently modified by chemical steps or additional bioconversion processes to synthetize final steroidal active pharmaceutical ingredients (APIs).

Regardless of the aforementioned approaches, steroid synthesis bioprocesses can be classified into three groups: (i) bioprocesses for production of steroidal intermediates from natural sterols, (ii) bioprocesses for modification and/or functionalization of steroidal molecules, (iii) *de novo* biosynthesis of steroids (Figure 2). A more detailed description of these bioprocesses is presented below, highlighting the most relevant achievements in the last decades, as well as outlining the trends and perspectives of research into this field. In no case, this review intends to carry out a comprehensive analysis of all the publications, patents and microbial strains described so far.

## Current achievements of steroid biotechnology

### Microbial production of steroidal intermediates from sterols

Natural sapogenins such as diosgenin have been used as basic precursors in the steroid chemistry for decades (Figure 1; Herráiz, 2017). For example, 16-dehydropregnenolone acetate derived from diosgenin can be converted to valuable steroids by chemical synthesis processes (Hanson, 2005; Laveaga, 2005). Diosgenin can be also transformed into steroidal intermediates with therapeutic activities by microbial biotransformation (Wang et al., 2007; Wang F. Q. et al., 2009; Wang W. et al., 2009). Nevertheless, the utilization of sapogenins as feedstock has been significantly reduced since it presents several drawbacks: high costs, multiple steps, low yields, and wild plant resources depletion (Tong and Dong, 2009; Wang et al., 2011). Therefore, sapogenins have been progressively replaced by several natural sterols (e.g., phytosterols, cholesterol) that can also be biotransformed into steroidal derivatives with properties similar to certain sex hormones (Figure 1). These steroidal derivatives, which are actually intermediates or byproducts of the sterol catabolic pathway (e.g., AD) (see below), are currently used as key precursors (synthons) for the chemical synthesis of steroid-based drugs such as corticosteroids, mineralocorticoids and oral contraceptives. Several types of phytosterols (e.g., soybean, pine, paper industry wastes) are used generally as industrial feedstock instead of cholesterol (obtained from animal fats and oils), due to the exhaustive quality controls required for the use of any type of animal basic precursor (Fernandes et al., 2003; Malaviya and Gomes, 2008).

**Figure 3:**
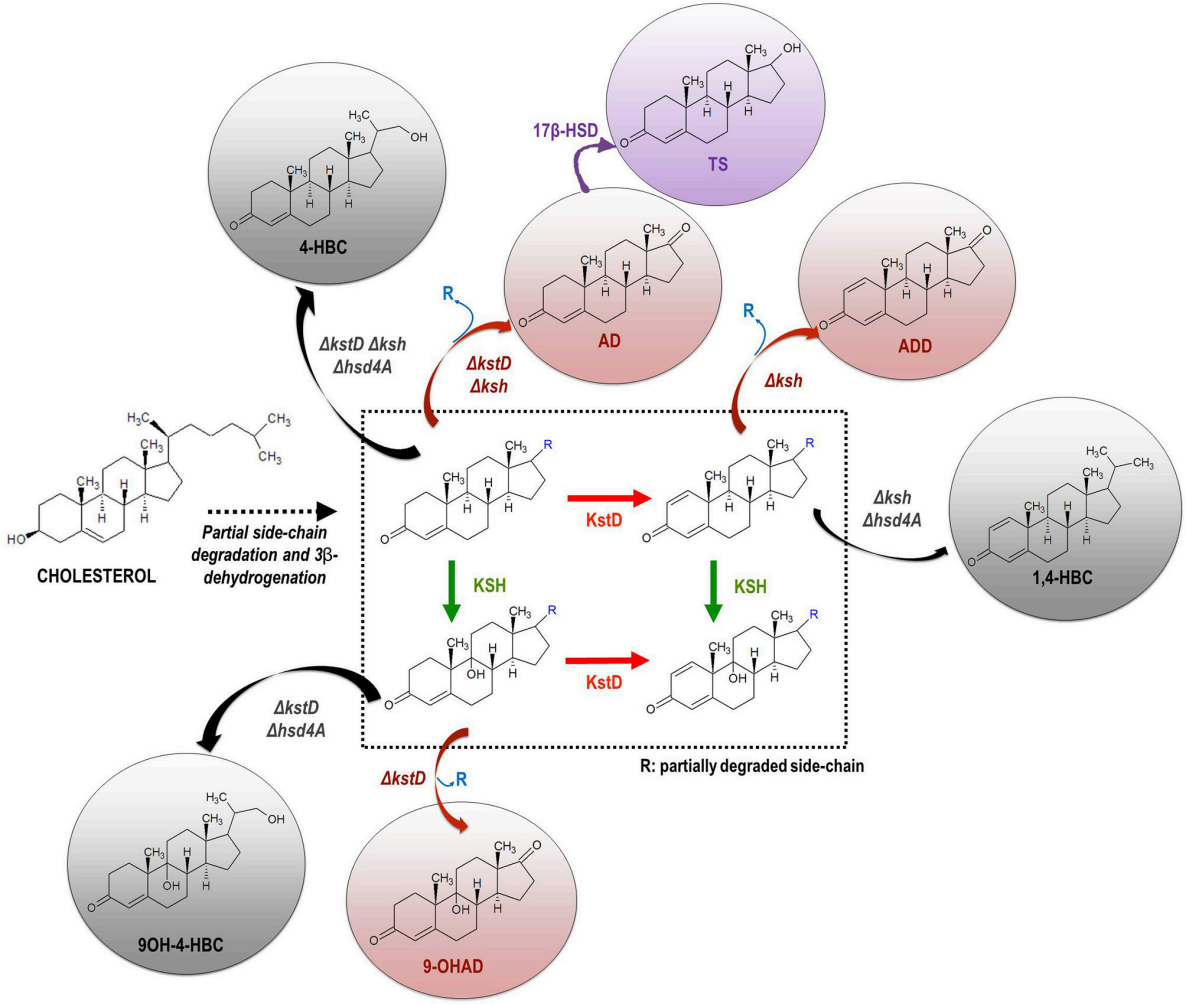
Scheme of the rational construction of mycobacterial strains producing steroidal intermediates from natural sterols by metabolic engineering approaches. Based on the knowledge of the catabolic pathway of sterols in actinobacteria, the redirection of metabolic flux toward the accumulation/synthesis of C-19 (red) or C-22 (gray) steroidal intermediates can be addressed via single/multiple gene deletions. The metabolic flux can be also redirected to the synthesis of new steroidal compounds by incorporating heterologous enzymatic activities (pink) (e.g., 17β-hydroxysteroid dehydrogenase, 17β-HSD). *kstD*, 3-ketosteroid-Δ^1^-dehydrogenase; *ksh*, 3-ketosteroid-9α-hydroxylase; *hsd4A*, hydroxysteroid dehydrogenase. Abbreviations of metabolites: ADD, 1,4-androstadiene-3,17-dione; AD, 4-androstene-3,17-dione; 9OH-AD, 9α-hydroxy-4-androstene-3,17-dione; TS, 17β-hydroxy-4-androstene-3,17-dione; testosterone; 1,4-HBC, 22-hydroxy-23,24-bisnorchol-1,4-diene-3-one; 4-HBC, 22-hydroxy-23,24-bisnorchol-4-ene-3-one; 9OH-4-HBC, 9,22-dihydroxy- 23,24-bisnorchol-4-en-3-one.

In general, actinobacteria producing mycolic acids such as those belonging to the genera *Gordonia, Mycobacterium, Nocardia*, and *Rhodococcus*, are able to metabolize cholesterol and other natural sterols (Donova, 2010; García et al., 2012; Galán et al., 2017a). In last decades numerous members of these genera have been isolated from different natural sources (e.g., soil) and subsequently optimized by conventional mutagenesis to select bacterial strains that produce steroidal intermediates of interest. For example, the C-19 steroids (e.g., AD, ADD, 9OH-AD, and TS), the C-22 steroids (e.g., 4-HBC, 1,4-HBC, 9OH-4-HBC) and the sitolactone intermediate are currently produced from sterols by microbial bioconversion (Figure 1). Interestingly, only strains of the genus *Mycobacterium* are used for production purposes at an industrial scale (Marsheck et al., 1972; Imada and Takahashi, 1980; Knight and Wovcha, 1981; Liu and Meng, 1981; Liu et al., 1994; Lo et al., 2002; Donova et al., 2005a,b; Wei et al., 2010a; Supplementary Table 1). New steroid-producing strains have also been rationally designed by metabolic engineering approaches in recent years (Table 1). Since certain C-19 steroids (e.g., AD, ADD, 9OH-AD) have been postulated as intermediates of the (chole)sterol catabolic pathway in actinobacteria (García et al., 2012; Galán et al., 2017a), redirecting metabolic fluxes toward their accumulation can be addressed by gene deletion and/or overexpression (Figure 3). For instance, the mutant strains of *Mycobacterium smegmatis* named MS6039 (Δ*MSMEG_6039*, Δ*kshB1*) and MS6039-5941 (Δ*MSMEG_6039* Δ*MSMEG_5941*, Δ*kshB1* Δ*kstD1*) mainly produced ADD and AD from sterols, respectively (Galán et al., 2017b). Several multiple-gene-deletion mutants of *M. neoaurum* ATCC 25795 were developed to produce 9OH-AD or C-22 derivatives from sterols (Yao et al., 2014; Xu et al., 2016). Furthermore, the overepression of the *kshA* gene in the 9OH-AD-producing mutant of *M. neoaurum* contributed greatly to improve the process selectivity by reducing the accumulation of undesirable byproducts (e.g., AD) that impair downstream purification processes (Yao et al., 2014). Wei et al. (2010b) also engineered an AD-producing mutant by deleting the *kstD* gene of *M. neoaurum* NwlB-01, which had been previously isolated from soil samples and selected because of its ability to accumulate mixtures of AD/ADD from phytosterols. Alternatively, Wei et al. (2014) improved the production of ADD in the strain NwlB-01 by the overexpression of various *kstD* genes from different sources. Similarly, Yuan et al. (2015) constructed a 9OH-AD-producing strain by overexpressing the *kshA* and *kshB* genes from *M. smegmatis* mc^2^155 and *Gordonia neofelifaecis*, respectively, in the strain *Mycobacterium* sp. NRRL B-3805 lacking 9α-hydroxylase activity. Finally, Fernández-Cabezón et al. (2017a) overexpressed two *17*β*-HSD* genes from different sources in *M. smegmatis* MS6039-59410 with the objective of producing testosterone from sterols in a single fermentation step.

**Table 1:** Microbial production of steroidal intermediates from natural sterols by recombinant DNA technology approaches.

**Parental Strain**	**Main product**	**Genetic manipulations**
*M. neoaurum* NwlB-01	ADD	Overexpression of an endogenous *kstD* gene or a heterologous *kstD* gene derived from the bacterium *Arthrobacter simplex* (Wei et al., 2010b, 2014)
*R. equi* USA-18	ADD	Deletion of the *kshB1* gene (Yeh et al., 2014)
*M. neoaurum* ATCC 25795	AD/ADD	Deletion of the *kshA1* and *kshA2* genes (strain named NwIB-XII) (Xu et al., 2016)
*M. smegmatis* mc^2^155	(1) AD (2) ADD	(1) Deletion of the *kshB1* and *kstD1* genes (strain named MS6039-5941); (2) Deletion of the *kshB1* gene (strain named MS6039) (Galán et al., 2017b)
*M. neoaurum* ATCC 25795	9OH-AD	Multiple deletion of several *kstD* genes and overexpression of an endogenous *kshA* gene (Yao et al., 2014)
*Mycobacterium* sp. NRRL B-3805	9OH-AD	Overexpression of the *kshA* and *kshB* genes of *M. smegmatis* mc^2^155 and *G. neofelifaecis* NRRL B-59395, respectively (Yuan et al., 2015)
*R. rhodochrous* DSM43269	1,4-HBC	Multiple deletion of all the *kshA* genes (Δ*ksA12345*) (Petrusma et al., 2011; Wilbrink et al., 2011)
*M. neoaurum* ATCC 25795	(1) 4-HBC (2) 1,4-HBC (3) 9OH-4-HBC	(1) Deletion of multiple *kshA* and *kstD* genes (Δ*kstD123*, Δ*kshA12*) and *hsd4A* gene*;* (2) Deletion of multiple *kshA* genes *(ΔkshA12*) and the *hsd4A* gene; (3) Deletion of multiple *kstD* genes *(ΔkstD123*) and the *hsd4A* gene (Xu et al., 2016)
*M. smegmatis* MS6039-5941 (Galán et al., 2017b)	TS	Overexpression of two genes encoding 17b-HSDs from *Comamonas testosteroni* and *Choliobolus lunatus* (Fernández-Cabezón et al., 2017a)

*Several examples of actinobacterial strains designed by Metabolic Engineering approaches for this purpose are shown. Main end products obtained at the bioconversions and no other by-products are indicated. ADD, 1,4-androstadiene-3,17-dione; AD, 4-androstene-3,17-dione; 9OH-AD, 9α-hydroxy-4-androstene-3,17-dione; TS, 17β-hydroxy-4-androstene-3,17-dione; testosterone; 1,4-HBC, 22-hydroxy-23,24-bisnorchol-1,4-diene-3-one; 4-HBC, 22-hydroxy-23,24-bisnorchol-4-ene-3-one; 9OH-4-HBC, 9,22-dihydroxy-23,24-bisnorchol-4-ene-3-one*.

Other authors have attempted to construct mutant strains producing steroidal intermediates from sterols in species of the genus *Rhodococcus*. However, the presence of multiple steroid catabolic pathways apparently convergent and the existence of basal levels of certain isoenzymes (e.g., KstD, KshA, or KshB) have hindered the development of stable producers in these species (van der Geize et al., 2000, 2001, 2002a,b, 2008; Petrusma et al., 2011; Fernández de las Heras et al., 2012; Liu et al., 2016; Guevara et al., 2017a,b). In certain members of the genus *Mycobacterium* such as *M. smegmatis, M. tuberculosis* or *M. neoaurum*, the deletion of a single key gene (*kshA, kshB*, or *kstD*) appears to be sufficient to prevent an efficient mineralization of sterols (Brzostek et al., 2005; Andor et al., 2006; Capyk et al., 2009; Brzezinska et al., 2013; Yao et al., 2014; Galán et al., 2017b). In contrast, the deletion of five *kshA* genes was needed to construct a mutant strain of *Rhodococcus rhodochrous* DSM43269 incapable of mineralizing sterols (Petrusma et al., 2011). Similarly, two *kstD* genes were deleted into *R. ruber* str. Chol-4 to avoid cholesterol mineralization (Fernández de las Heras et al., 2012). So far, only Yeh et al. (2014) were able to design an ADD-producing strain of *Rhodococcus equi* by deleting only one *kshB* gene. Moreover, the equivalent mutants of the genus *Mycobacterium* and *Rhodococcus* accumulate different types and/or amounts of steroidal intermediates from sterols. For instance, AD/ADD-producing mycobacterial strains accumulate small amounts of 4-HBC/1,4-HBC alcohols, whereas the respective mutants of *Rhodococcus* accumulate notable amounts of their acid derivatives 3-oxo-23,24-bisnorchol-4-en-22-oic acid (4-BNC) and 3-oxo-23,24-bisnorchol-1,4-dien-22-oic acid (1,4-BNC) (Marsheck et al., 1972; Wilbrink et al., 2011; Yeh et al., 2014; Xu et al., 2016; Galán et al., 2017b). For example, Wilbrink et al. (2011) constructed a mutant strain of *R. rhodochrous* DSM 43269 (strain RG32) devoid of 9α-hydroxylase activity, which accumulated mostly 1,4-BNC and only small amounts of ADD from sterols.

The successful implementation of the aforementioned metabolic engineering approaches would not have been possible without the numerous studies of catabolic pathways of steroids developed in various model actinobacteria (e.g., *M. smegmatis* mc^2^155, *R. jostii* RHA1, *M. tuberculosis* H37Rv), the development of new molecular biology tools for genetic manipulation of these non-model bacteria and the sequencing and annotation of their genomes (Kendall et al., 2007, 2010; van der Geize et al., 2007; Mohn et al., 2012; Uhía et al., 2012; Haußmann et al., 2013; Li et al., 2014; Bergstrand et al., 2016; Crowe et al., 2017).

### Microbial functionalization of steroidal molecules

**Figure 4:**
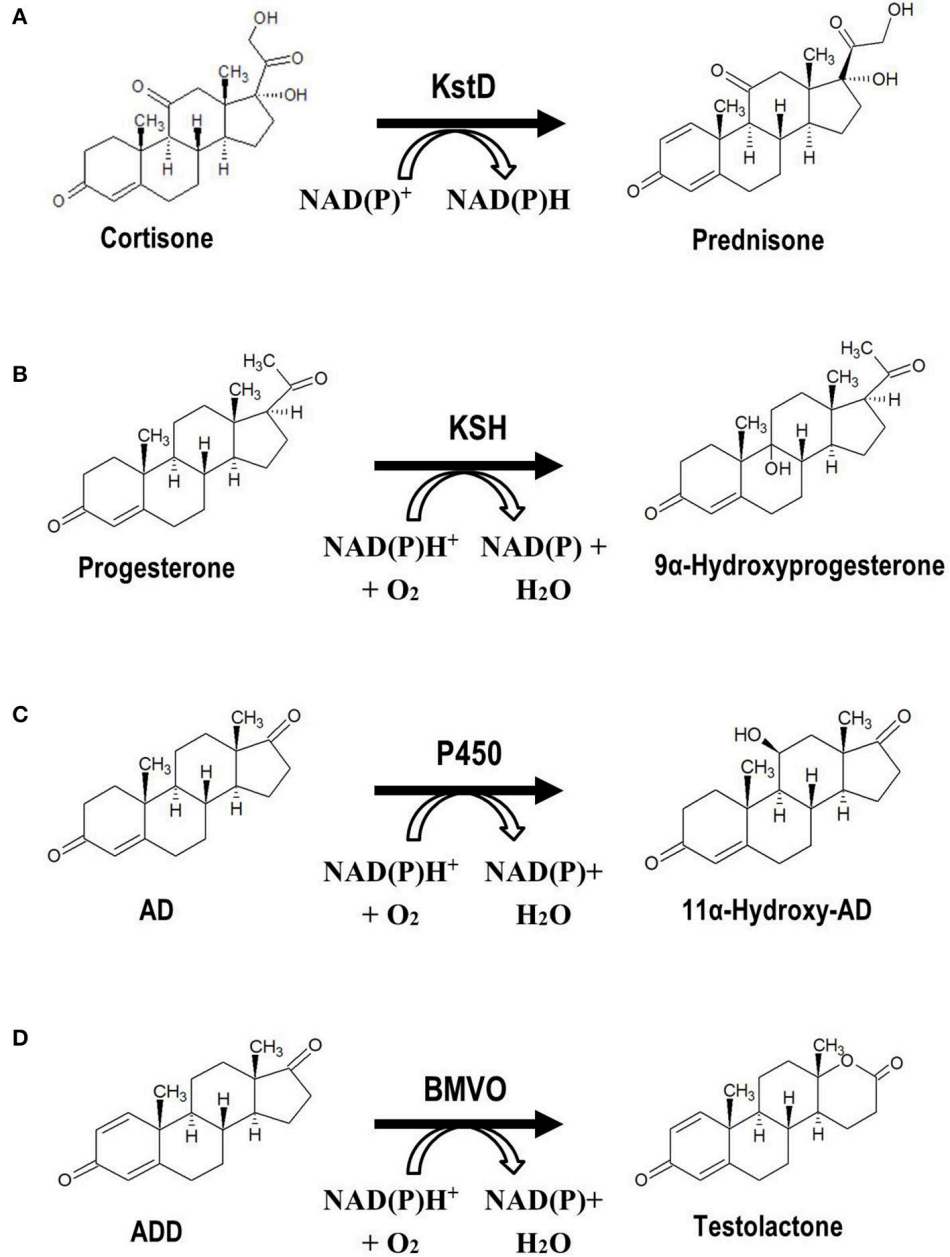
Microbial functionalization of steroidal molecules of industrial relevance. **(A)** Δ1-Dehydrogenation of 3-ketosteroids: conversion of cortisone into prednisone. **(B)** 9α-Hydroxylation of 3-ketosteroids: conversion of progesterone into 9α-hydroxy-progesterone. **(C)** 11α-Hydroxylation of 3-ketosteroids: conversion de AD into 11α-hydroxy-AD. **(D)** Baeyer-Villiger oxidation: conversion of ADD into testolactone. KstD, 3-ketosteroid-Δ^1^-dehydrogenase, KSH, 3-ketosteroid-9α-hydroxylase, P450, 11α-hydroxylase, cytochrome P450, BMVO, Baeyer-Villiger monooxygenase.

The chemical modification of steroidal molecules has attracted considerable attention in last decades. Baeyer-Villiger oxidations, hydroxylations, and reduction-oxidation (redox) reactions are some of the most relevant modifications (functionalizations) performed on steroidal compounds by chemical synthesis processes. These and other functionalizations of steroids can alternatively be performed by biocatalytic procedures. For example, redox reactions such as oxidations of alcohols, reductions of carbonyl groups, dehydrogenations of C-C bonds or hydrogenations of C-C double bonds, can be catalyzed by specific enzymes. The Δ1,2-dehydrogenation and the 17β-reduction of 3-ketosteroids are two examples of enzymatic redox reactions of relevance for steroid synthesis (Figure 4). Hydroxylations of steroidal molecules, in which a hydrogen atom is replaced by a hydroxyl group (e.g., 7α, 9α-, 11α-, 11β-, 16α-, and 17α-hydroxylation), can also be catalyzed by several families of hydroxylase enzymes such as multicomponent cytochromes P450 and monooxygenases (Figure 4). Even the classical Baeyer-Villiger oxidations, in which an ester/lactone is generated from a ketone/cyclic ketone (e.g., transformation of ADD into testolactone, an aromatase inhibitor) (Kołek et al., 2008), can be catalyzed by enzymes called Baeyer-Villiger monooxygenases (BVMOs) that use molecular oxygen and NADPH (Figure 4; Leisch et al., 2011). To perform these chemical functionalizations on steroidal molecules, several wild-type microorganisms that possess the steroid-modifying enzymatic activities of interest are frequently used at an industrial scale (Supplementary Table 2; Zhang et al., 1998; El-Kadi and Mostafa, 2004; Li et al., 2005, 2017; Reese, 2007; Roglič et al., 2007; Manosroi et al., 2008; Yildirim et al., 2010; Peart et al., 2011; Bhatti and Khera, 2012; Donova and Egorova, 2012; Prakash and Bajaj, 2017). Since these bioconversions often exhibit various drawbacks such as low selectivity and substrate conversion yields, and may even require the culture of opportunistic pathogenic microorganisms (e.g., *Rhizopus oryzae*), it has been proposed the design of alternative MCFs by using recombinant DNA technologies in recent years. Several model microorganisms such as *Escherichia coli, Bacillus subtilis*, or *Saccharomyces cervisiae* have been chosen to heterologously express one or more genes encoding the proteins involved in the single enzymatic steps of interest mentioned above (i.e., 9α-hydroxylation and Δ1,2-dehydrogenation of 3-ketosteroids) (Figure 4, Table 2; Andor et al., 2006; Arnell et al., 2007; Li et al., 2007; Petrusma et al., 2009; Zhang W. et al., 2013; Zhang et al., 2016; Shao et al., 2017). Non-conventional yeasts and bacteria (e.g., *Schizosaccharomyces pombe, Pseudomonas putida*) have been also used for this purpose (Ruijssenaars et al., 2007; Hakki et al., 2008; Petrič et al., 2010 Table 2). To our knowledge, none of the bioprocesses designed by using recombinant DNA technologies has been industrially implemented due to the low achieved substrate conversions that seem to be the consequence of an inefficient transport of steroids. For this reason, the rational genetic improvement of microorganisms capable of efficiently transporting and modifying steroids may be a challenging approach to follow. Zhang W. et al. (2013) increased successfully the 3-ketosteroid-Δ^1^-dehydrogenase activity of an *Arthrobacter simplex* strain by genomic integration of multiple copies of the endogenous *ksdD* gene under the control of a constitutive promoter, reaching with the recombinant strain a conversion yield of substrate 32.9% higher than that of wild-type strain. Similarly, Kiss et al. (2015) increased the endogenous 15β-hydroxylase activity in *B. megaterium* by overexpressing the cytochrome CYP106A2.

**Table 2:** Microbial functionalization of steroidal molecules by recombinant DNA technology approaches.

**Substrate**	**Main product**	**Microorganism**	**Genetic manipulation**
AD	ADD	*B. subtilis* WB600	Overexpression of the *ksdD* gene from *A. simplex* AS1.94 (Li et al., 2007)
AD	ADD	*B. subtilis* 168	Overexpression of the *ksdD* gene from *M. neoaurum* JC-12 (Zhang W. et al., 2013)
AD	ADD	*B. subtilis* 168	Hyperproduction of the KsdD protein from *M. neoaurum* JC-12 (gene codon optimization). Overexpression of the *katA* gene from *Bacillus pumilus* ML 413 encoding a catalase to reduce the endogenous levels of H_2_O_2_ (Shao et al., 2017)
AD	ADD	*R. ruber* str. Chol-4	Multiple deletion of several *kshA* genes *(ΔkshA123*) or single deletion of *kshB* gene *(ΔkshB*) (Guevara et al., 2017b)
Cortisone acetate	Prednisone acetate	*A. simplex* 156	Overexpression of the endogenous *ksdD* gene by integrating multiple gene copies under the control of a constitutive promoter (Zhang H. et al., 2013)
AD	9OH-AD	*E. coli* BL21	Overexpression of the *kshA1* gene from *M. smegmatis* mc^2^155 (Andor et al., 2006)
AD	9OH-AD	*E. coli* BL21	Overexpression of the *kshA* and *kshB* genes from *R. rhodochrous* DSM 43269 (Petrusma et al., 2009)
AD	9OH-AD	*B. subtilis* 168	Overexpression of the *kshA* gene from *M. neoaurum* JC-12 and the endogenous *gdh* gene encoding a 1-dehydrogenase glucose to regenerate NADH (Zhang et al., 2016)
AD	9OH-AD	*R. ruber* str. Chol-4	Multiple deletion of several *kstD* genes *(ΔkstD123*) (Fernández de las Heras et al., 2012)
Progesterone	9α-Hydroxy-progesterone	*E. coli* BL21	Overexpression of the *kshA1* gene from *M smegmatis* mc^2^155 (Arnell et al., 2007)
AD	Testosterone	*M. smegmatis* mc^2^155	Overexpression of a gene encoding a 17b-HSDs from *Comamonas testosteroni* or *Cochliobolus lunatus* (Fernández-Cabezón et al., 2017a)
DHEA	7α-Hydroxy-DHEA	*S. cerevisiae*	Overexpression of the gene encoding cytochrome CYP7B (Vico et al., 2002)
Progesterone	11α-Hydroxy-progesterone	*Schizosaccharomyces pombe* 1445	Hyperproduction of 11 α-hydroxylase from *R. oryzae* (co-expression of all constituents: cytochrome CYP509C12 and NAD(P)H reductase) (Petrič et al., 2010)
11-Deoxycortisol	Hydrocortisone	*S. pombe*	Hyperproduction of the human 11β-hydroxylase (co-expression of all constituents: cytochrome CYP11B1, adrenoxin and adrenoxin reductase) (Hakki et al., 2008)
11-Deoxycorticosterone	15β-Hydroxy-deoxycorticosterone	*E. coli*	Hyperproduction of 15β-hydroxylase from *B. megaterium* (coexpression of all constituents: cytochrome P450, adrenoxin and adrenoxin reductase) (Hannemann et al., 2006)
Testosterone	15β-Hydroxy-testosterone	*P. putida* S12	Overexpression of the gene encoding cytochrome CYP106A2 from *B. megaterium* (Ruijssenaars et al., 2007)
Cyproterone acetate	15β- Hydroxy-cyproterone acetate	*B. megaterium* ATCC 13368	Overexpression of the endogenous gene encoding cytochrome CYP106A2 (15β-hydroxylase) (Kiss et al., 2015)
Progesterone	17α-Hydroxy-progesterone	*Pichia pastoris* GS115	Overexpression of the gene encoding human cytochrome P45017 (17α-hydroxylase) (Kolar et al., 2007)
(1) DHEA (2) PREG (3) C1	(1) AD (2) PROG (3) C2	*C. glutamicum* R31	Overexpression of the 3β-hydroxysteroid dehydrogenase/ isomerase gene (*MSMEG_5228*) from *M smegmatis* mc^2^155 (García-Fernández et al., 2017)

*Several examples of recombinant strains designed to modify stereo- and regio-specifically steroidal molecules of pharmacological interest are shown. Main end products obtained at the bioconversions and not other by-products identified are indicated. ADD, 1,4-androstadiene-3,17-dione; AD, 4-androstene-3,17-dione; 9OH-AD, 9α-hydroxy-4-androstene-3,17-dione; DHEA, 3β-hydroxy-5-androstene-7-one; dehydroepiandrosterone; C1, 5-pregnen-16α,17α-epoxy-16β-methyl-3β-ol-20-one; epoxymethylpregnenolone; C2, 4-pregnen-16α,17α-epoxy-16β-methyl-3,20-dione; epoxymethylprogesterone*.

Alternatively, several authors have developed high-yield bioconversions of steroids based on *in vitro* enzymatic processes that use recombinant proteins purified or obtained as cleared cell lysate and that frequently incorporate a couple enzyme to recycle the cofactor consumed in the reaction (i.e., NAD(P)/H) (Fogal et al., 2013; Hoebenreich et al., 2017). None of these bioprocesses has been implemented industrially to date due to the high production cost of active recombinant enzymes.

### *De novo* biosynthesis of steroids in yeasts

Several microbial bioprocesses designed by synthetic biology and system biology approaches have been successfully implemented for the industrial production of high-added value compounds (Lee et al., 2016; Nielsen and Keasling, 2016; Silber et al., 2016). However, steroid industry has hardly explored this path, and there are only two examples that highlight the potential of these disciplines. *De novo* biosynthesis of progesterone and hydrocortisone from simple carbon sources (e.g., galactose, ethanol) was successfully achieved in recombinant strains of *Saccharomyces cerevisiae*, by engineering the endogenous sterol biosynthesis pathway to generate a cholesterol-like molecule that served as a precursor to a multi-enzymatic heterologous route mimicking human steroid biosynthesis (Duport et al., 1998; Szczebara et al., 2003).

## Current challenges of steroid biotechnology

### Constructing a second generation of mycobacterial strains

The economic feasibility of certain industrial bioprocesses for steroid production is limited by low sterol conversion yields and low product selectivity achieved with the currently available *Mycobacterium* strains. Consequently, the construction of a second generation of MCFs with improved properties is one of the most relevant short-term challenges for the production of steroidal intermediates from sterols. By implementing the knowledge related to the bacterial steroid catabolism acquired in recent years and a combination of empirical methodologies proposed below, new more robust industrial bioprocesses may be designed.

In last years the genome sequencing and annotation of several industrial mycobacterial strains obtained by traditional approaches has been reported (e.g., *Mycobacterium* sp. NRRL B-3805, *Mycobacterium* sp. NRRL B-3683, *Mycobacterium* sp. VKM Ac-1815D) (Bragin et al., 2013; Shtratnikova et al., 2014, 2015; Rodríguez-García et al., 2016). The mutations present in these industrial strains have also been determined with the aim of identifying potential genetic targets for a rational design of new highly-producer MCFs. Complementary approaches to conventional mutagenesis such as the Adaptive Laboratory Evolution (ALE) can be applied for this same purpose. The ALE methodology has already been implemented to optimize numerous bioprocesses over the last 25 years (Dragosits and Mattanovich, 2013). Taking into account that overproduction of compounds and high stress tolerances are determined by interaction of multiple parameters/factors, that are not always known and predictable and therefore cannot be easily addressed through rational approaches, the ALE methodology based on long-term adaptation of microbial strains can be an interesting alternative. By progressively increasing the sterol concentration of the medium in a chemostat or in several consecutive batch cultures, ALE evolved strains that efficiently biotransform sterols into steroidal intermediates could be selected (Mondaca et al., 2017). In addition, ALE methodology could be combined with reverse engineering strategies, which seek to “rationalize” the selected phenotypes by identifying the mutations present in the evolved strains and improve them, if possible, in new rounds of evolution.

A better understanding of the steroid catabolic complexity may be also key to optimizing several current industrial bioprocesses in actinobacteria. The partial degradation of products described in many mycobacterial strains could be avoided through the elimination of undesirable enzymatic activities via rational gene deletion. Yao et al. (2014) demonstrated that two residual enzymatic activities KstD (KstD2 and KstD3) present in the mutant Mut_*MN*−*KSTD*1_ (Δ*kstD1*) of *M. neoaurum* ATCC 25795 markedly decreased the conversion yield of 9OH-AD from sterols. Interestingly, KstD2 and KstD3 enzymes are encoded by genes located in a second steroid gene cluster that is homologous to the C-19+ cluster recently described in *M. smegmatis*, which has apparently evolved independently from the upper cholesterol *kstR*-regulon and appears to be necessary for the efficient metabolization of C-19 steroids in this bacterium (i.e., AD, ADD, 9OH-AD) (Fernández-Cabezón et al., 2017b). Certain industrial mycobacterial strains such as *M. neoaurum* VKM Ac-1815D and *Mycobacterium* sp. NRRL B-3805 do not have the C-19+ gene cluster. In fact, a genomic deletion of this cluster is found when their genomes are compared to the genome of the close phylogenetic strain *M. neoaurum* DSM 44074 (ATCC 25795). Therefore, the deletion of the C-19+ gene cluster or of certain genes located therein (e.g., *kstD, kshA, kshB*), may increase the bioconversion yields of those producer strains derived from *M. smegmatis, M. neoaurum* or other mycobacteria carrying the C-19+ cluster. On the other hand, the identification of enzymes involved in each of the steps of sterol catabolism may be useful for the construction of new strains producing new steroidal intermediates of industrial interest (e.g., 4-HBC, 1,4-HBC, 9-OH-4-HBC) (Xu et al., 2016), as well as in the determination of limiting enzymatic steps leading to low conversion yields and accumulation of undesirable by-products. For example, a deep understanding of the enzymatic steps involved into the sterol side-chain degradation could be useful for the design of new AD/ADD-producing strains that do not accumulate the intermediates 4-HBC/1,4-HBC. In order to further understand the regulatory mechanisms of sterol catabolism and steroid production, several transcriptomic and proteomic studies have been also done in recent years, increasingly focused on higher-producer strains (Liu et al., 2016a,b; Xiong et al., 2017a,b).

The characterization of the C-19+ gene cluster above mentioned, as well as of other steroid degradation gene clusters in bacteria, has allowed to identify multiple steroid-modifying isoenzymes with different catalytic properties (e.g., substrate specificity, cellular localization) (Knol et al., 2008; Petrusma et al., 2011, 2012; Penfield et al., 2014; Guevara et al., 2017a,b; Wang et al., 2017), that should be considered to design more efficient industrial bioprocesses. For example, the selective production of ADD from sterols in a single fermentation step remains a challenge at an industrial scale. To date, all available mycobacterial strains accumulate mixtures AD/ADD that hamper the subsequent purification processes (Marsheck et al., 1972; Wei et al., 2010b; Galán et al., 2017b). Several hypotheses have been raised about the impossibility of achieving bioconversions close to 100%: (i) existence of certain enzymatic activities 1-en reductase that catalyze the reverse reaction (Goren et al., 1983; Egorova et al., 2002); (ii) phenomena of inhibition and/or toxicity because of the ADD accumulation (Smith et al., 1993); (iii) insufficient levels of enzymatic activity KstD (Wei et al., 2014). On the basis of this latter hypothesis, several authors have overexpressed endogenous or heterologous *kstD* genes (involved into the sterol catabolism) in ADD-producing strains that have led to remarkable improvements in bioconversion selectivity but still insufficient (Wei et al., 2010b, 2014). Surprisingly, the utilization of alternative *kstD* genes involved into different steroid catabolic pathways or even a combination of *kstD* genes have not been explored yet. For instance, the simultaneous overexpression of the *kstD1* (*MSMEG_5941*) and *kstD2* (*MSMEG_2869*) genes from *M. smegmatis* involved into the cholesterol and C-19 steroids catabolism, respectively, could be investigated in ADD-producing strains with the aim of favoring the Δ1,2-dehydrogenation reactions in different steps (on different substrates) during the sterol bioconversion (Supplementary Figure 1).

Finally, the construction of new MCFs is necessary for the direct conversion of sterols into steroidal compounds that are not part of the steroid catabolism in bacteria (e.g., 11α-OHAD, 11β-OHAD, 17β-OHAD). As mentioned above, most of these compounds are currently synthesized in a second fermentation step from C-19 or C-22 steroidal intermediates. The need for a second fermentation involves further purification steps of intermediate products and a significant increase in the time and cost of the overall process. Therefore, the development of one-step bioprocesses by incorporating new steroid-modifying enzymatic activities in certain mycobacterial strains may be a convenient approach. For example, the overexpression of several heterologous genes encoding 11α-hydroxylases in the AD-producing mutant *M. smegmatis* MS6039-5941 have been already proposed to synthesize 11α-OHAD from (phyto)sterols in a one-step fermentation (Ríos et al., 2017). However, given that most of these enzymatic reactions are catalyzed by membrane-associated multicomponent cytochromes P450 which are very abundant in their native microorganisms (e.g., certain species of fungi have among several tens to a hundred of cytochromes P450), significant efforts will be required to identify the genes encoding the enzymatic activities of interest, as well as to achieve an adequate production of active enzyme that allows redirecting fully (or mostly) the metabolic flow toward the formation of steroidal product.

## Microbial functionalization of steroids

The challenges proposed above are based on the utilization of mycobacterial species as MCFs. However, it is likely that the biosynthesis of a broad portfolio of steroids cannot be achieved by using a single microbial *chassis* as MCF. Therefore, the identification of alternative microbial *chassis*, i.e., alternative microorganisms with desirable properties (e.g., efficient steroid transport, high stress tolerance, non-pathogenicity, absence of undesired steroid-modifying enzymatic activities, availability of multiple genetic modification tools), must be investigated. For example, the utilization of the well-known industrial actinobacteria *Corynebacterium glutamicum* has been recently applied for designing new functionalization bioprocesses of steroids (García-Fernández et al., 2017).

On the other hand, the identification of novel steroid-modifying enzymes is also required for the design of new bioprocesses. To do this, several approaches such as the exploration of alternative ecological niches (e.g., marine, anoxic and thermopile environments) by means of metagenomic approaches, the annotation of genomes available in database, the development of new proteins by protein engineering and the implementation of high-throughput assays for strain screening can be applied. For example, Ribeiro et al. (2017) have investigated the identification of thermostable enzymes for developing *in vitro* bioconversions of steroids, since thermostability can increase not only the enzyme half-life but also the solubility of steroids notably improving reaction yields.

### *De novo* biosynthesis of steroids in actinobacteria

Most of the limitations observed in current industrial bioprocesses are directly related to the intrinsic properties of steroidal molecules (e.g., low solubility in aqueous media, high cell toxicity). With the aim of overcoming these limitations, different technological approaches such as micronization or emulsification with surfactants of steroidal substrates, utilization of solvent organics in two-phase systems, cell immobilization and development of in continuous or *in situ* production systems have been investigated in recent years (de Carvalho et al., 2004; Donova et al., 2007; Egorova et al., 2009; Lam, 2010; Marques et al., 2010; Donova and Egorova, 2012; Galán et al., 2017a).

Alternatively, the development of MCFs capable of *de novo* synthesizing steroids from conventional feedstock such as glucose has been proposed. In this sense, it was mentioned previously that the *de novo* biosynthesis of progesterone and hydrocortisone was successfully achieved in recombinant strains of *S. cerevisiae* (Duport et al., 1998; Szczebara et al., 2003). Similarly, *de novo* biosynthesis of steroids in mycobacteria might be also achieved (Supplementary Figure 2). Briefly, taking account that steroids are part of the isoprenoid family that are synthesized in all organisms through a modular synthesis pattern, it is possible to channel the metabolic flux of the universal isoprenoid precursors (i.e., isopentenyl pyrophosphate, IPP; dimethylallyl pyrophosphate, DMAPP) toward the synthesis of squalene through a limited number of heterologous enzymatic steps and, from this compound, to generate by cyclization the steroidal molecule lanosterol. Thus, it is possible to assemble an artificial biosynthetic route of sterols with a partially-interrupted cholesterol catabolic pathway to synthesize steroidal intermediates of interest. The bioconversion of lanosterol into steroidal intermediates of interest by *Mycobacterium* sp. NRRL B-3805 has been already described (Wang et al., 1995). Nevertheless, for a successful implementation on an industrial scale of the approaches detailed here, new genetic tools must be designed. In recent years significant advances have been made in the development of genome-editing tools (e.g., recombineering-assisted by CRISPR-Cas9), which facilitate the construction of microbial strains for different purposes such as the industrial applications of White Biotechnology (Pines et al., 2015; Jakočiunas et al., 2016). These tools allow the simultaneous introduction of several types of genomic modifications, shortening the time of strain development and avoiding the use of selection markers. At present, a recombineering system for the genomic modification of mycobacteria is available (Murphy et al., 2015) and a CRISPR-Cas9 interference system (CRISPRi) has been successfully implemented (Choudhary et al., 2015; Rock et al., 2017), which opens the possibility of designing even more versatile genome editing systems in mycobacteria (e.g., recombineering assisted by CRISPR-Cas9). For instance, Yan et al. (2017) have recently developed a recombineering assisted by CRISPR-Cas12a (Cpf1) (i.e., a new type of CRISPR-Cas system), that allows an efficient editing of *M. smegmatis* genome. Moreover, in the next few years, additional efforts must be also made to construct promoter libraries of different strength for fine-tuned gene expression in mycobacteria (Kanno et al., 2016), as well as the engineering of regulatory circuits for an optimal control of the heterologous gene expression (Bradley et al., 2016).

## Concluding remarks

**Figure 5:**
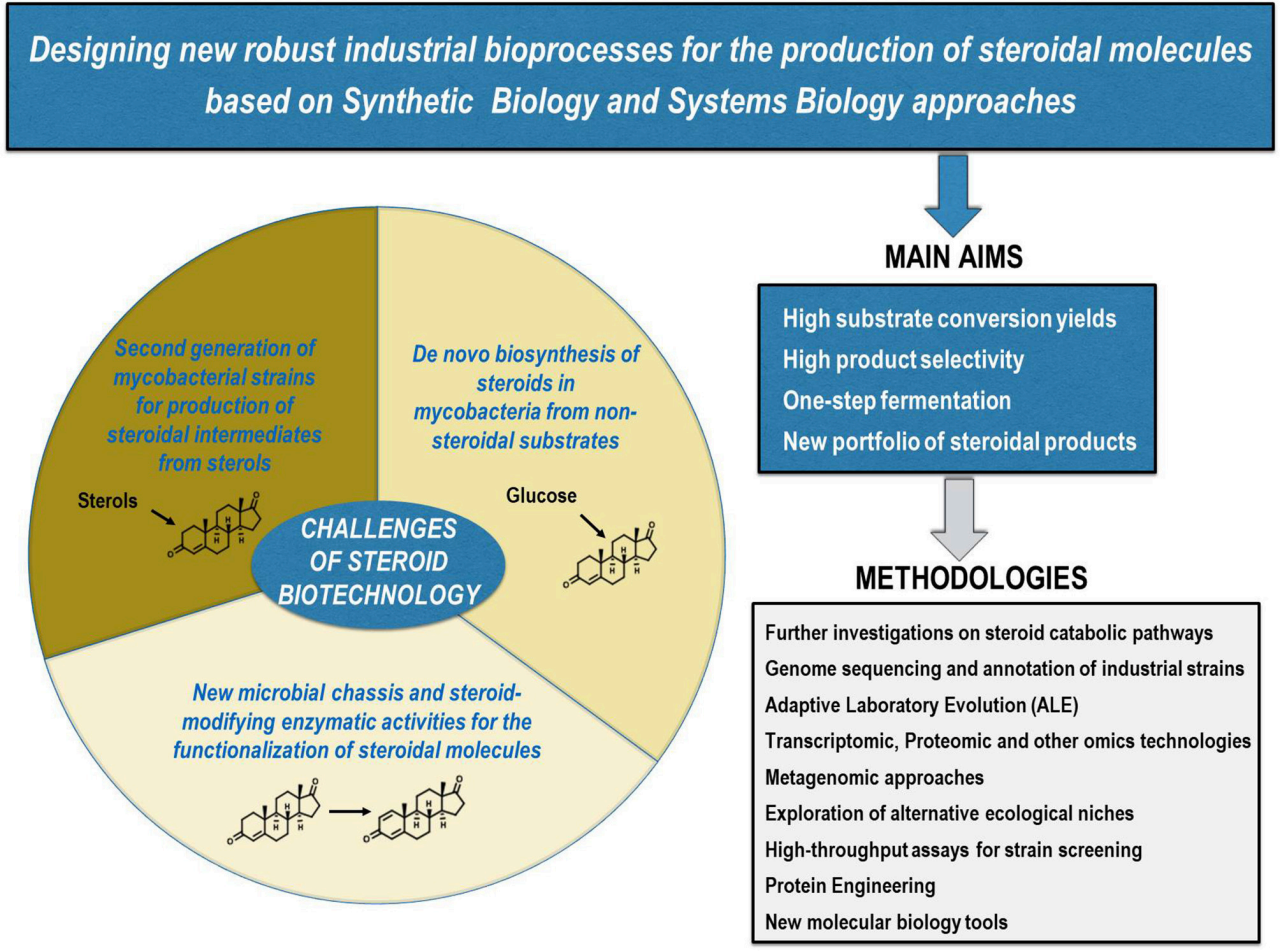
Current challenges of steroid biotechnology. New microbial cell factories are needed to design new industrial bioprocesses for the production of steroids à *la carte*, i.e., more robust one-step biotechnological processes with higher substrate conversion yields and product selectivity. Using multiple currently available methodologies, at least the following challenges should be addressed: (i) construction of a second generation of mycobacterial strains for the production of steroidal intermediates from sterols; (ii) identification of new microbial *chassis* and steroid-modifying enzymatic activities for the functionalization of steroidal molecules; (iii) *de novo* biosynthesis of steroids from non-steroidal substrates in mycobacteria.

The biotechnological production of steroids as other industrial bioprocesses, such as the production of amino acids or antibiotics, has been traditionally based on the isolation of microorganisms of interest and their subsequent improvement through physicochemical mutagenesis and selection. Although in recent years the optimization of several bioprocesses has been addressed through recombinant DNA technology approaches, these techniques have not been hardly applied for steroid synthesis (Lee et al., 2016; Nielsen and Keasling, 2016; Silber et al., 2016). This fact could be in part explained by the intrinsic difficulties associated with the utilization of steroids (e.g., low solubility in aqueous media, high cell toxicity) and the difficult genetic manipulation and bad reputation of the MCFs commonly used in these bioprocesses (i.e., mycobacterial species). Nevertheless, this trend is changing as we have showed in this review highlighting the most relevant research carried out on steroid biotechnology based on recombinant DNA technologies. In our opinion, the main challenge to become competitive with the current industrial chemical processes is to design more robust bioprocesses with higher substrate conversion yields and product selectivity. An additional challenge will be to offer a new portfolio of steroids with the ultimate goal of producing steroids à *la carte*. To achieve all these challenges, it will be necessary to construct new MCFs based on the implementation of Synthetic Biology and Systems Biology approaches (Figure 5). The long road that must be undertaken to design bio-sustainable processes such as those proposed here is justified by the growing demand for steroidal pharmaceuticals and the commitment increasingly needed with the environment.

## Author contributions

LF-C and JG: Conceived and designed the work; LF-C, JG, and BG: prepared the figures, and wrote the manuscript; JG: Ensured the financial and material resources; LF-C, BG, and JG: Reviewed the final draft.

### Conflict of interest statement

The authors declare that the research was conducted in the absence of any commercial or financial relationships that could be construed as a potential conflict of interest.
