# Supplementary material for: New Insights on Steroid Biotechnology
Source: Front Microbiol. 2018 May 15;9:958. doi: 10.3389/fmicb.2018.00958 (PMC5962712; doi:10.3389/fmicb.2018.00958)
Supplement: Supplementary file 1 [file Table_1.DOCX]

**Supplementary Table 1.** Microbial production of steroidal intermediates from natural sterols by traditional approaches. Several relevant industrial strains isolated and improved by conventional mutagenesis processes for this purpose are shown. Only main end-products obtained at the bioconversions but not others by-products identified are indicated. Abbreviations: ADD (1,4-androstadiene-3,17-dione), AD (4-androstene-3,17-dione), 9OH-AD (9α-hydroxy-4-androstene-3,17-dione), TS (17β-hydroxy-4-androstene-3,17-dione; testosterone), 1,4-HBC (22-hydroxy-23,24-bisnorchol-1,4-diene-3-one) and sitolactone ((4aR,6aS,9aS,9bS)-6a-methyloctahydrocyclopenta[f]chromene-3,7(2H,8H)-dione).

| STRAIN | MAIN PRODUCT | ORIGIN/ PHENOTYPIC DESCRIPTION |
| --- | --- | --- |
| *Mycobacterium* sp*.* NRRL B- 3683 | ADD | Physical mutagenesis of *Mycobacterium* sp. isolated from soil samples with the ability to accumulate ADD from sterols (Marsheck et al., 1972) |
| *Mycobacterium* sp. VKM Ac-1816D | ADD | Unknown source. Strain available at All-Russian Collection of Microorganisms (VKM IBPM RAS) (Donova et al., 2007) |
| *M. neoaurum* NwlB-01 | AD/ADD | Strain isolated from soil samples that produces a mixture of steroid intermediates from sterols (Wei et al., 2010a) |
| *Mycobacterium* sp*.* NRRL B-3805 | AD | Physical mutagenesis of *Mycobacterium* sp. NRRL B-3683 followed by the selection of an AD-producing mutant (Marsheck et al., 1972) |
| *Mycobacterium* sp*.* VKM Ac-1815D | AD | Unknown source. Strain available at All-Russian Collection of Microorganisms (VKM IBPM RAS) (Egorova et al., 2002) |
| *Mycobacterium* sp. 2-4M | 9OH-AD | Physico-chemical mutagenesis of *Mycobacterium* sp. VKM Ac-1815D and selection of 9OH-AD-producing mutants (Donova et al., 2005a) |
| *Mycobacterium* sp. VKM Ac-1817D | 9OH-AD | Available from All-Russian Collection of Microorganisms (VKM IBPM RAS) (Donova et al., 2005b) |
| *Mycobacterium* sp. NRRL B-3805 | TS | Application of alternative fermentation conditions to favour the production of TS (instead of AD) from sterols (Liu et al.,1994) |
| *Mycobacterium* sp. ST2 | TS | Chemical mutagenesis of *Mycobacterium* sp*.* NRRL B-3805 and selection of TS-producing mutants (Lo et al., 2002) |
| *Mycobacterium* sp. VKM Ac-1816D | TS | Application of alternative fermentation conditions to favour the production of TS (instead of ADD) from sterols (Egorova et al., 2009) |
| *Mycobacterium* sp*.* NRRL B- 11389 | 1,4-HBC | Physical mutagenesis of *Mycobacterium parafortuitum complex* ATCC 25790 (*Mycobacterium neoaurum* ATCC 25790) and selection of a 1,4-HBC-producing mutant (Imada and Takahashi, 1980) |
| *M. fortuitum.* NRRL B-8128 | Sitolactone | Chemical mutagenesis of *Mycobacterium* sp*.* ATCC 6842 and selection of sitolactone-producing mutants (Knight and Wovcha, 1981) |
| *M. fortuitum.* NK-XHX-103 | Sitolactone | Unknown source. Strain available at China Center for Type Culture Collection (No. of CCTCC M2013543) (Liu and Meng, 1981) |
