# Supplementary material for: New Insights on Steroid Biotechnology
Source: Front Microbiol. 2018 May 15;9:958. doi: 10.3389/fmicb.2018.00958 (PMC5962712; doi:10.3389/fmicb.2018.00958)
Supplement: Supplementary file 2 [file Table_2.DOCX]

**Supplementary Table 2.** Microbial functionalization of steroidal molecules by traditional approaches. Several examples of microbial strains isolated and improved conventionally to modify stereo- and regio-specifically steroidal molecules of pharmacological interest are shown. Only main end-products obtained at the bioconversions but not others by-products identified are indicated. Abbreviations: ADD (1,4-androstadiene-3,17-dione), AD (4-androstene-3,17-dione), 9OH-AD (9α-hydroxy-4-androstene-3,17-dione), DHEA (3β-hydroxy-5-androstene-7-one; dehydroepiandrosterone).

| Substrate | Main Product | Microorganism |
| --- | --- | --- |
| Cortisone acetate | Prednisone acetate | *Arthrobacter simplex* (Zhang et al., 1998) |
| AD | ADD | *A. simplex* ATCC 6946 (Prakash and Bajaj, 2017) |
| AD | 9OH-AD | *Rhodococcus equi* VKM Ac- 953*, Rhodococcus rhodochrous* VKM Ac-1282 (Donova, 2010) |
| Pregnenolone | 7α-Hydroxypregenelone | *Fusarium oxysporum* var. cubense (Reese, 2007) |
| DHEA | 7α-Hydroxy-DHEA/  7β-Hydroxy-DHEA | *Mucor racemosus* (Li et al., 2005) |
| DHEA | 7α,15α-Dihydroxy-DHEA | *Colletotrichum lini* (Li et al., 2017) |
| Progesterone | 11α-Hydroxyprogesterone | *Rizhopus nigricans* ATCC 6227b (Roglič et al., 2007) |
| Progesterone | 11β-Hydroxyprogesterone | *Trichoderma harzianum, Trichoderma hamatum* (El-Kadi and Mostafa, 2004) |
| Testosterone | 14α-Hydroxytestosterone | *Aspergillus wentii* MRC 200316 (Yildirim et al., 2010) |
| (1)Testosterone;  (2) Progesterone | 1. 15α-Hydroxytestosterone; 2. 15α-Hydroxyprogesterone | *F. oxysporum* var. cubense (Reese, 2007) |
| Androsta-4,6-diene-3,17-dione | 16β-Hydroxyandrosta-4,6-diene-3,17-dione | *F. oxysporum* var. cubense UAMH 9013 (Peart et al., 2011) |
| Progesterone | 17α-Hydroxyprogesterone | *Bacillus sphaericus (*ATCC 245*,* ATCC 7063*,* ATCC 13805*,* TISTR 670) and *A. simplex* ATCC 6946 (Manosroi et al., 2008b) |
| ADD | Testololactone | *Purpureocillium lilacinum* AM111 (Kolek et al., 2008) |
