# Supplementary figures and images for: New Insights on Steroid Biotechnology
Source: Front Microbiol. 2018 May 15;9:958. doi: 10.3389/fmicb.2018.00958 (PMC5962712; doi:10.3389/fmicb.2018.00958)

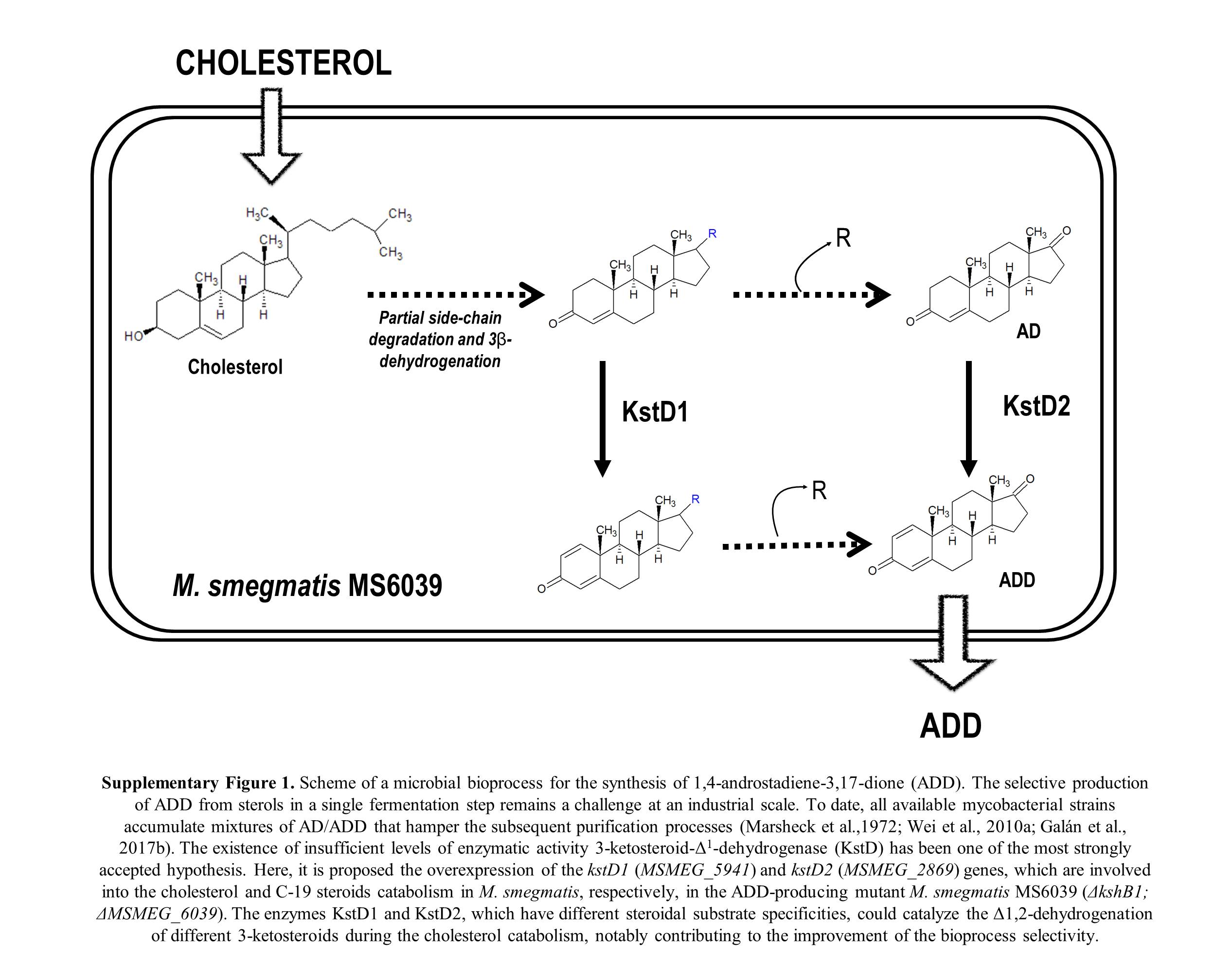

Supplement: Supplementary file 3 [file Image_1.JPEG]

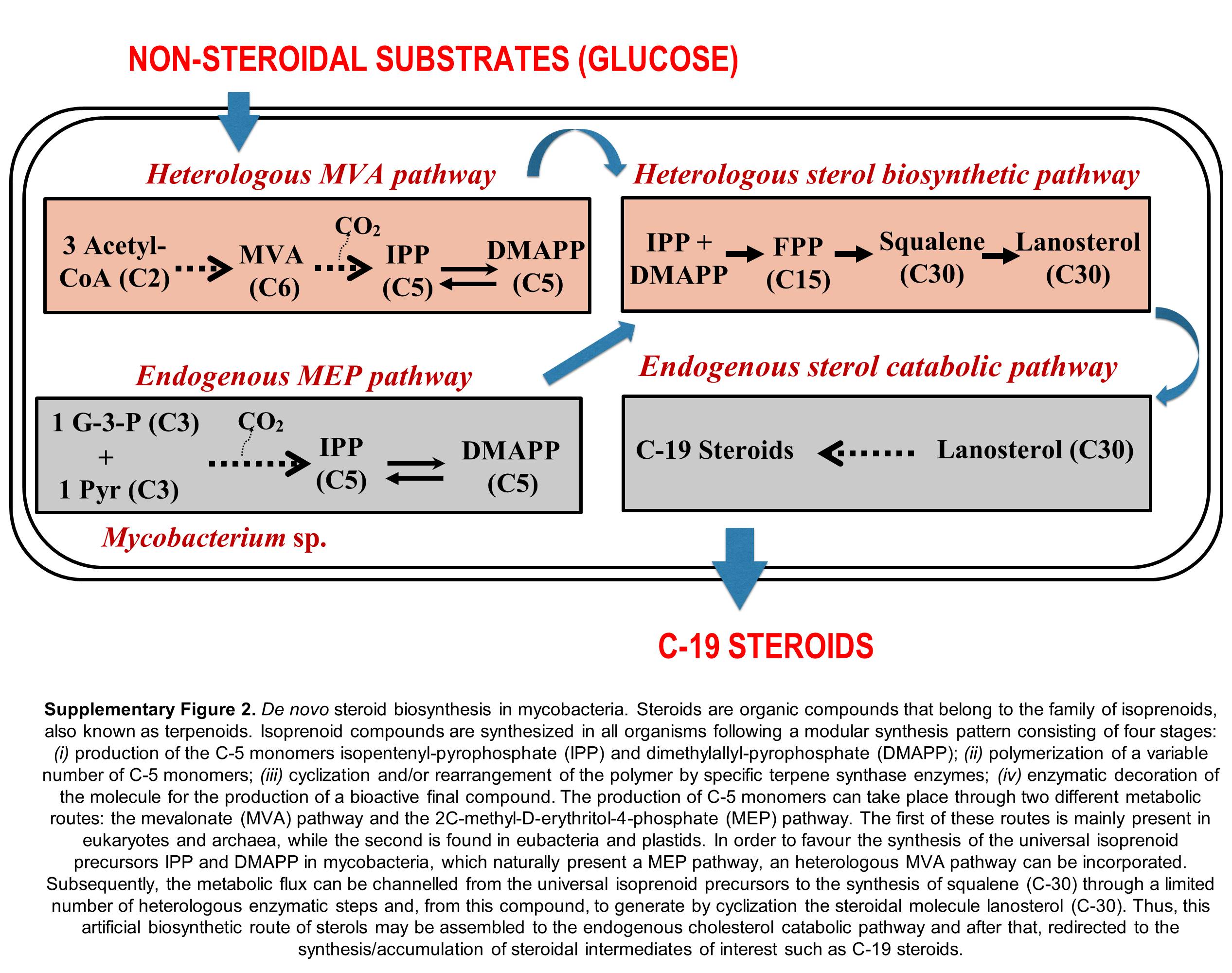

Supplement: Supplementary file 4 [file Image_2.JPEG]
